# Low Total Dose of Anti-Human T-Lymphocyte Globulin (ATG) Guarantees a Good Glomerular Filtration Rate after Liver Transplant in Recipients with Pretransplant Renal Dysfunction

**DOI:** 10.1155/2018/1672621

**Published:** 2018-08-16

**Authors:** Cristina Dopazo, Ramón Charco, Mireia Caralt, Elizabeth Pando, José Luis Lázaro, Concepción Gómez-Gavara, Lluis Castells, Itxarone Bilbao

**Affiliations:** ^1^Department of HPB Surgery and Transplants, Hospital Universitario Vall d'Hebron, Universidad Autónoma de Barcelona, Barcelona, Spain; ^2^Hepatology Unit, Department of Internal Medicine, Hospital Vall d'Hebron, CIBERehd, Universidad Autónoma de Barcelona, Barcelona, Spain

## Abstract

We aimed to evaluate the safety and efficacy of low doses of anti-T-lymphocyte globulin (ATG)-based immunosuppression in preserving renal function and preventing liver rejection in liver transplant (LT) recipients with pretransplant renal dysfunction. We designed a prospective single-center cohort study analyzing patients with pre-LT renal dysfunction defined as eGFR<60 mL/min/1.73m^2^, who underwent induction therapy with ATG (*ATG group, n=20)*. This group was compared with a similar retrospective cohort treated with basiliximab (*BAS group, n=20*). An economic analysis between both induction therapies was also undertaken. In the* ATG group, *45% and 50% of patients had recovered their renal function without acute cellular rejection (ACR) episodes at day 7 and 1 month after LT, respectively, versus 40% and 55% of patients in* the BAS group* (p=1). Renal function improved in both groups over time and no differences between groups were observed regarding one-year eGRF and one-year probability of ACR. Cost per patient of the ATG course was 403€ (r: 126-756) versus 2,524€ of the basiliximab course (p=0.001). In conclusion, induction with low dose of ATG or basiliximab in patients with pretransplant renal dysfunction is a good strategy for preserving posttransplant renal function; however the use of low-dose ATG resulted in a substantial reduction in drug costs. This trail is registered with* ClinicalTrials.gov number: *NCT01453218.

## 1. Introduction

Renal dysfunction in liver transplantation (LT) is one of the major concerns hindering posttransplant patient management and determining worse prognosis [1–3].

Renal dysfunction in cirrhotic patients is of multiple well-known causes [3–5]. According to published data, approximately 30% of cirrhotic patients on the waiting list for LT have some degree of renal impairment [6]. After LT, impaired renal function tends to recover partially or completely unless advanced parenchymatous lesions are significantly involved as a major cause of the dysfunction [7–12].

In this scenario, the feasibility of delaying the introduction of calcineurin inhibitors (CNI) in patients with a high risk of immediate posttransplant renal dysfunction, as in critically ill patients with severe ascites, hepatorenal syndrome, or pre-LT renal dysfunction in whom it would be desirable to allow their renal function to return to normal before the introduction of nephrotoxic CNI as part of a maintenance immunosuppressive regimen has already been demonstrated [13–20]. This practice is usually accompanied by induction immunosuppression therapy consisting of a chimeric monoclonal interleukin-2-receptor (CD25 antigen) antibody administered on day 0 and day 4 after LT.

Anti-human T-lymphocyte globulin (ATG) is an alternative to interleukin-2-receptor antagonistic induction therapy, with greater immunosuppressive power but higher hematologic and infectious adverse event rates widely reported in renal transplantation [20–25]. For this reason, induction therapies using polyclonal anti-thymocyte globulins in LT are not universally used since the liver is assumed to be less immunogenic than kidney grafts.

Considering the lower immunogenicity of the liver, we designed a study to evaluate the safety and efficacy of an immunosuppressor regimen plus induction therapy with low-dose ATG in preserving renal function and preventing liver rejection in LT recipients with pretransplant renal dysfunction.

## 2. Methods

### 2.1. Study Design

A prospective single-center cohort study of adult LT recipients with a pretransplant renal dysfunction (*ATG group)* was designed to evaluate the efficacy and safety of induction therapy with ATG plus steroids and tacrolimus (TAC). Pre-LT renal dysfunction was defined as an estimated glomerular filtration rate (eGFR) < 60 mL/min/1.73m^2^ under the MDRD4 formula on the day of LT. 

*Inclusion Criteria. *Adult patients on the waiting list for LT from brain-dead donors with pre-LT renal dysfunction were included. In cases of cirrhosis due to hepatitis C virus (HCV) infection, negative HCV-RNA was required. 

*Exclusion Criteria.* Exclusion criteria included retransplantation, multiorgan transplantation, acute liver failure, severe leucopenia (<1.2x10E9/L), and/or thrombocytopenia (<50x10E9/L).

Patients in the ATG study group were compared with a historical cohort of patients with pretransplant renal dysfunction (eGFR < 60 mL/min/1.73m^2^ under the MDRD4 formula on the day of LT), who underwent LT and received monoclonal interleukin-2-receptor (basiliximab) as induction therapy (*BAS group*). For every ATG patient, we retrospectively selected 1 age (+/-10 years), sex, diagnosis, and MELD score matched patients for comparison (1:1 matching).

The study was conducted in compliance with the provisions of the Declaration of Helsinki and Good Clinical Practice guidelines. This study was approved by the Hospital Vall d'Hebron Institutional Review Board (Barcelona, Spain). All patients provided their written informed consent form prior to initiation of the study and were allowed to withdraw at any time. The trial was registered with ClinicalTrials.gov number NCT01453218.

### 2.2. Immunosuppression with ATG Induction

Patients in the* ATG group *received induction therapy with anti-human T-lymphocyte globulin (Grafalon; Neovii Biotech GMBH; Germany). ATG was intravenously (i.v.) administered on Intensive Care Unit (ICU) admission at a dose of 1mg/kg/bodyweight. All patients were premedicated with methylprednisolone 250mg i.v., dexchlorpheniramine 5mg i.v., and paracetamol 1g. Following doses were given on days 2 and 4 with dose adjustment according to CD2/CD3 levels (>20cel/*μ*L). The third dose of ATG on day 4 was omitted if CD2/CD3 levels were below 20 cel/*μ*L and platelet counts < 50,000cells/mm^3^ on the day after the second dose. CD2/CD3 levels were also measured on days 7 and 14 after LT.

TAC initiation was delayed until at least the second day depending on urine output of more than 50ml/h and if improvement in eGFR was observed (≥ 30 mL/min/1.73m^2^). TAC was introduced at a low dose (0.05mg/Kg twice daily) and dosage adjustments were made to achieve a 12-hour trough level of 5 to 8 ng/dL during the first 3 months and less than 5 ng/dL thereafter if no rejection occurred.

Methylprednisolone was started at ICU admission coinciding with ATG premedication at doses of 250mg i.v., followed by 200mg i.v. per day, tapered to 20mg orally per day over 6 days. During follow-up, methylprednisolone was reduced to 16mg orally per day at the 4^th^ week, tapered to minimum doses for the following three months, and discontinued in all patients with normal liver function, except those with autoimmune disease.

Mycophenolate mofetil (MMF) at a dose of 1g twice a day was introduced on day 7 if TAC trough level failed to reach an adequate level and platelet count was > 50x10E9/L.

### 2.3. Immunosuppression with Basiliximab Induction

Patients in the* BAS group* received induction therapy with basiliximab (Simulect; Novartis, Basel, Switzerland) 20mg intravenously on day 0 intraoperatively after allograft reperfusion and on day 4 after LT.

The initiation of low TAC doses followed the same criteria as in the* ATG group. *Methylprednisolone 500 mg i.v. was administered intraoperatively, followed by 200mg i.v. per day, decreasing to 20mg orally per day over 6 days. During follow-up, methylprednisolone was reduced according to the same protocol as in the ATG group.

MMF at doses of 1g twice a day was started from day 1 if the platelet count was > 50x10E9/L.

### 2.4. Cytomegalovirus (CMV) and Pneumocystis Carinii Prophylaxis

All patients with a high risk for cytomegalovirus (CMV) infection (donor-positive, recipient-negative) received at least 3 months' prophylaxis with valganciclovir. CMV viral load was monitored weekly by PCR during the first month after transplant and monthly thereafter.

Additionally, pneumocystis carinii prophylaxis with trimethoprim and sulfamethoxazole or pentamidine was mandatory for all patients for at least 6 months.

### 2.5. Acute Cellular Rejection (ACR) Treatment

All suspected ACR episodes were proven by biopsy (BPAR) and stratified according to BANFF criteria [26]: indeterminate (portal inflammatory infiltrate that failed to meet the criteria for the diagnosis of ACR), mild (rejection infiltrate in a minority of triads that is generally mild and confined within the portal spaces), moderate (rejection infiltrate expanding most or all of the triads), and severe (moderate plus spillover into periportal areas and moderate to severe perivenular inflammation that extends into the hepatic parenchyma and is associated with perivenular hepatocyte necrosis). Treatment included 3 boluses of methylprednisolone (500 mg i.v.) if episodes were moderate or severe or increased doses of TAC if mild.

### 2.6. Endpoints

The primary efficacy endpoint was the combination of absence of ACR episodes and eGFR ≥ 60 mL/min/1.73m^2^ at day 7 and 1 month after transplantation. Secondary endpoints were one-year patient and graft survival, incidence of infections including CMV (PCR>1000 copies/ *μ*L), and the incidence of adverse events directly associated with ATG focused mainly on hematologic events (leucopenia and thrombocytopenia).

Demographic and baseline data of the recipients, donors, and surgical procedure were prospectively recorded in a database. During post-LT follow-up, documentation of clinical signs and laboratory data were obtained at baseline, days 7 and 14, and months 1, 3, 6, 9, and 12.

Follow-up was one year.

### 2.7. Cost Study

A financial analysis was also made to compare the impact of ATG induction therapy with that of our standard treatment with basiliximab. The analysis was based on the cost of the number of doses administered.

### 2.8. Statistical Analysis

This was an exploratory study and sample size determination was not based on statistical power. A preanalysis was conducted with 40 subjects (20 in each arm) and the behavior of this group was considered the population estimate.

Categorical variables were summarized as counts and percentages and continuous variables as medians with range. Group comparisons were made by the Mann–Whitney test for continuous data and chi-square test with Fisher's correction for categorical data. The Friedman test was used to detect differences among different values of one variable. Time to reach ACR was calculated with the Kaplan Meier method using the log-rank test for treatment comparisons.

In order to increase statistical power, a primary combined endpoint was used as a single dichotomous outcome. The composite endpoint was considered when BPAR was absent and eGFR ≥ 60 mL/min/1.73m^2^ was present.

Differences were considered statistically significant when p <0.05. Statistical analysis was performed using IBM SPSS Statistics 23.0 software.

## 3. Results

From January 2012 to December 2016, twenty patients received ATG as immunosuppression induction therapy. They were compared with 20 matched patients who received basiliximab immunosuppression induction therapy from January 2005 to December 2011. No differences were found between groups regarding age, sex, primary liver disease, comorbidities, and MELD; however, significant differences were observed regarding pre-LT eGFR between groups. No patients were on hemodialysis at the time of LT* (see Table 1).*

### 3.1. ATG Dosage

Median first ATG dose was 74±10mg. Thirteen (65%) patients received a second dose, mean 79±7mg, and four patients (20%) received a third dose, mean of 78±16mg.

CD2/CD3 levels dropped to a median of 70 cel/ *μ*L (r: 10-297) after the first ATG dose and to 28 cel/ *μ*L (r: 0-240) after the second. Fourteen days after LT, CD2/CD3 levels had returned to normal range [median 330 cel/ *μ*L (49-1350)].

### 3.2. Basiliximab Dosage

All patients in the* BAS group* received the two doses of 20 mg i.v. of basiliximab at day 0 and day 4 after LT.

### 3.3. CNI Administration

**Figure 1:**
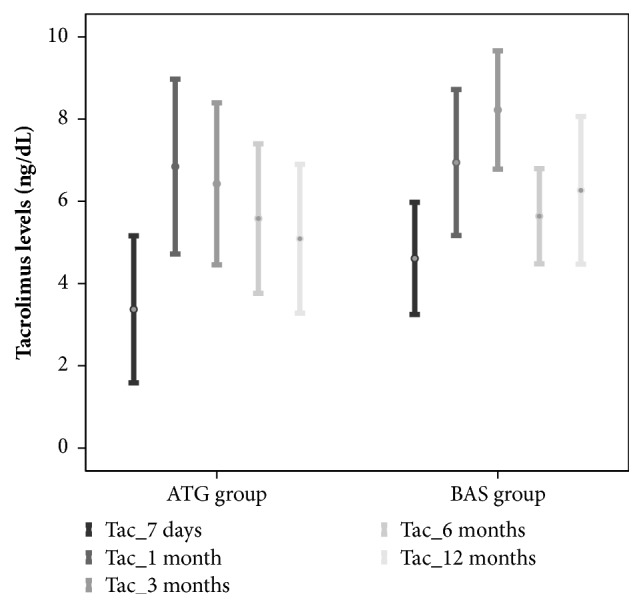
Tacrolimus levels after liver transplantation.

The introduction of TAC was delayed a mean of 5±2 days in the* ATG group* compared to a mean of 2±0.5 days in the* BAS group* (p=0.001). No differences were found in mean TAC levels between groups at day 7 after LT [3 ng/dL (r: 1-8) in the* ATG group* versus 5 ng/dL (r: 1-9) in the* BAS group, *p=0.29], not even at one, 3, 6, and 12-months after transplant. See*Figure 1*.

### 3.4. Endpoints

#### 3.4.1. Primary Combined Endpoint

Efficacy of primary combined endpoints had been achieved in 45% and 50% of patients at day 7 and 1 month after LT, respectively, in the* ATG group* versus 40% and 55% of patients at day 7 and 1 month after LT, respectively, in* the BAS group* (p=1).

#### 3.4.2. Renal Function

Ten of 20 patients (50%) had recovered their renal function (eGFR ≥60 mL/min/1.73m^2^) at day 7 after LT, continuing with the same percentage 1 month after LT in the ATG group. Eight of 20 patients (40%) and 11 of 20 patients (55%) had recovered their renal function (eGFR ≥60 mL/min/1.73m^2^) at day 7 and 1 month after LT, respectively, in the BAS group; these differences were not significant between groups.

**Figure 2:**
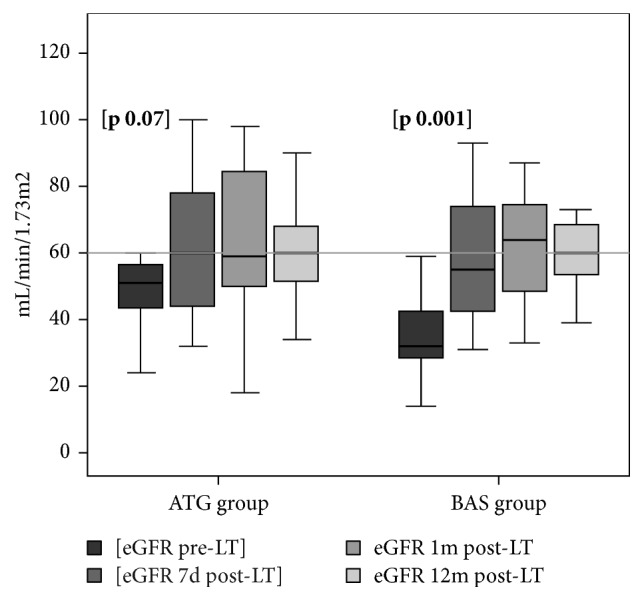
Glomerular filtration rate between groups at one year.

Evolution of eGFR is shown in*Figure 2*. An improvement in renal function was observed over time in both groups, being only significant at 7 days after LT compared to before LT in BAS group.

No differences were observed during follow-up and renal function at one year after LT was 58±16 mL/min/1.73m^2^ in the* ATG group* versus 62±16 mL/min/1.73m^2^ in the* BAS group* (p=0.31).

#### 3.4.3. ACR Episodes

ACR had occurred in 2 patients (10%) in the ATG group and none in the BAS group at day 7 after LT (p= 0.48). No more ACR episodes were observed in either group up to the end of the first month after LT.

**Figure 3:**
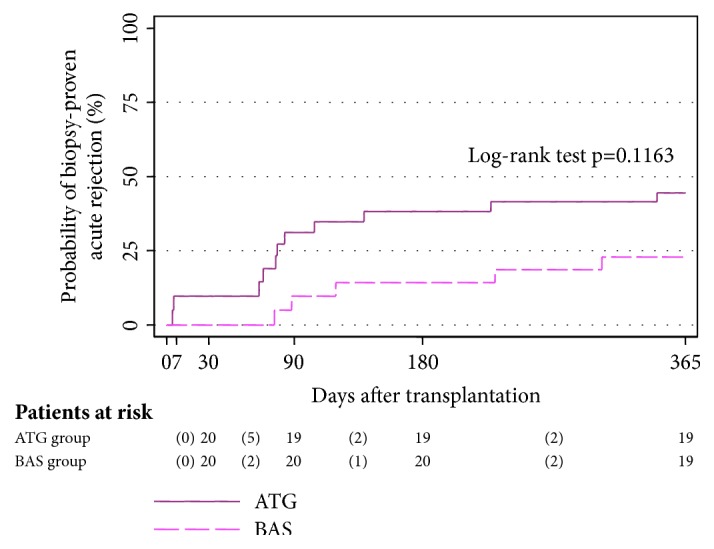
Cumulative probability of biopsy-proven rejection after transplantation.

Although the probability of BPAR was 2-fold higher in the* ATG group* compared with the BAS group, these differences were not significant (Figure 3). Eight patients (40%) in the* ATG group* presented some ACR episode during follow-up: 4 were moderate and 4 mild. ACR was reported in four patients (20%) in the* BAS group: *2 were moderate and 2 mild. All cases in both groups were successfully treated with boluses of methylprednisolone and/or increased doses of TAC. There were no episodes of severe ACR.

#### 3.4.4. Secondary Endpoints

Regarding mortality only two patients died during follow-up, one in each group. One in the* ATG group* was due to biliary complications related to hepatic artery thrombosis and further sepsis 2 months after LT. The other was a 69-year-old patient who died from decompensated cirrhosis due to chronic rejection 11 months after LT. TAC had to be withdrawn at day 28 owing to severe neurologic symptoms; however ductopenia appeared in the liver biopsy over 6 months later and the patient was treated with methylprednisolone, mTOR, and reintroduction of TAC. No clinical and pathologic response occurred.

No patients underwent retransplantation during follow-up, leading to 1-year graft and patient survival of 95% (*Table 2*).

No differences in the infection rate were observed between groups. Results of secondary endpoints at the end of follow-up are shown in*Table 2*.

### 3.5. Costs

The use of low-dose ATG resulted in a substantial reduction in drug costs compared to basiliximab. The* ATG group* received a median dose of 1.96 mg/kg (r: 0.65-4.16) and a median total dose of 160 mg (r: 50-300). Using a whole-sale acquisition cost for a 100-mg vial of ATG (Grafalon; Neovii Biotech GMBH; Germany) (252€) at our facility, the median drug cost for a course/patient of ATG induction was 403€ (r:126-756) versus 2,524 € per patient in the* BAS group* (p=0.001).

## 4. Discussion

This study demonstrated that induction therapy based on low-dose ATG preserves renal function in cirrhotic patients undergoing LT with pretransplant renal dysfunction.

ATG induction has been widely used in kidney transplantation. Results in this setting revealed fewer ACR episodes and less delayed graft function. Studies are divided into those that use a standard course (1.5mg/Kg for five to six doses) [21–24] and those that use a short course (1.5g/Kg for three to five doses) [27, 28] showing the same benefits and less degree of leucopenia and thrombocytopenia.

The use of any antibody therapy for induction in liver transplantation remains controversial [29–31]. The liver is considered an immunologically privileged organ and the use of antibodies to prevent rejection has been perceived as unnecessary and may increase the risk of overimmunosuppression. In a five-year randomized prospective study published by Boillot et al. [31] in the pre-MELD era, ATG induction in LT failed to exert any beneficial effect on rejection prevention and patient and graft survival.

However, the role of ATG induction in LT has been revisited in recent years and seems to provide the same benefits using a short-course therapy, permitting delayed CNI introduction at low doses to avoid CNI-induced renal impairment [17, 18].

Previous studies in LT reported a low ACR rate and renal function recovery in the early posttransplant period in patients at high risk of acute renal failure using variable doses of ATG induction therapy, around 1mg/kg - 2mg/kg per day over 3 days [15–19]. More recently, Yoo et al. [20] published their experience in the largest series including 500 patients who underwent a steroid-free protocol with ATG induction given at 3mg/Kg divided into two doses of 1.5mg/kg. They obtained excellent results with an ACR rate of 23% in five-year follow-up, good outcomes, low complication rates, and good renal function preservation. Moreover, Montenovo et al. [25] retrospectively compared, for the first time, the clinical effects of ATG versus basiliximab as induction therapies in LT in a population with normal pretransplant renal function. The ATG was administered at 1.5mg/kg/day over 3 days with delayed TAC introduction. Their results showed a significantly lower ACR rate in favor of ATG (18% versus 27%) and decreased creatinine levels in both groups in a median follow-up of 5 years; however, no data on eGFR were reported.

In concordance with these results, our study showed delayed introduction of reduced-dose TAC under the protection of induction therapy based on low doses of ATG in patients with pretransplant renal dysfunction to be associated with a low ACR rate in the first month after LT and renal function recovery with no increase in the infection rate. The fact that the use of polyclonal antibodies did not increase the risk of infections in our series may be related to the low total median ATG dose used (1.96 mg/kg), lower than reported by other authors [17–19].

The most significant finding in our study was that approximately half of the patients treated with either of the induction therapies already had normal renal function with no rejection episodes at one week after LT. Results one month later were similar. The main causes of renal function recovery were good function of the new liver and the delay in CNI initiation. Thus, rationale for using ATG or basiliximab is to increase the immunosuppressive effect meanwhile to prevent ACR until renal function improves, and not because they have a direct effect. It is important to point out that although the number of patients who achieved normal renal function was the same in both groups at one week after LT, TAC was introduced significantly later in the ATG group. The reason was that the authors assumed the more immunosuppressive effect to be associated with the polyclonal antibodies, and thus overimmunosuppression by TAC addition was avoided. However, rejection occurred in 10% of patients in the ATG group during the first month after LT, a rate similar to that reported in other studies [20–25]. Improvement in renal function was observed in both groups over time, with no significant differences at one year after LT.

The disappointing result in our series was that the probability of rejection was double in the ATG group at the end of the study compared with the BAS group. These differences were not significant, probably due to the small number of patients in each group. We cannot rule out the possibility that a higher total ATG dose might improve these results; however, we should point out that the ACR rate remained low during the first month after transplant. Nevertheless, all ACR episodes were mild or moderate and none were corticoid-resistant.

Regarding the safety of ATG administration, it was well tolerated and only 4 patients presented thrombocytopenia or leucopenia which was easily managed by dose reduction or interruption, with platelets and leucocytes being in normal range at one month after transplant, similar to report by other studies [18–20]. Moreover, excellent one-year patient and graft survival were reported in both groups and neither of the two deaths during follow-up could be attributed to the induction therapy.

The greatest benefit of the use of low-dose ATG as induction therapy was the significant financial saving due to the direct cost of the drug compared to basiliximab. The average saving was more than 2,000€ per LT patient. The literature reported two economic analyses in the kidney transplant setting. Marfo et al. [28] compared the clinical and economic impact of using short-course versus standard-course ATG, with no significant differences in acute rejection episodes and a significant cost reduction using the short course. Recently, Cremashi et al. [32] compared quality-adjusted life years (QALYs) between ATG and basiliximab, with a modest increase in QALYs and lower long-term costs in the ATG cohort. However, no data on liver transplantation were reported.

The major limitations of this study were the low number of patients owing to the exploratory nature of the trial and bias in inclusion criteria. Patients with severe thrombocytopenia or leucopenia were not included in the ATG group, which probably selected patients with less portal hypertension and who were less critically ill compared with the BAS group. This became evident as shown by the higher pretransplant eGFR and lower MELD score in the ATG group. Despite that, not differences in outcomes were observed between groups.

In summary, induction therapy with low doses of ATG or anti-IL2 antagonists in cirrhotic patients with pretransplant renal dysfunction are good strategies for preserving posttransplant renal function, with the cost of ATG being much lower. Owing to a direct effect of ATG on platelets and leucocytes, induction with these antibodies should not be recommended in patients with severe thrombocytopenia or leucopenia, findings that are more frequent in very advanced cirrhosis with severe portal hypertension. Nevertheless, it should be taken into account that our study included a small number of patients, and thus, prospective, randomized, control studies are required to confirm these results.

## Figures and Tables

**Table 1:** Patient characteristics and surgical data.

	**ATG Group **(n=20)	**BAS group **(n=20)	**p-value**
**Age (years)**	60(±6)	57 (±7)	0.143
**Male, n (%)**	18 (90%)	17 (85%)	1
**Primary liver disease**			0.215
Alcoholic	11 (55%)	11(55%)	
Hepatitis C	4 (20%)	8 (40%)	
HCC	3 (15%)	1 (5%)	
Hepatitis B	1 (5%)	-	
NASH	1 (5%)	-	
**Pre-LT Arterial Hypertension, n (%)**	6 (30%)	5 (25%)	0.723
**Pre-LT Diabetes Mellitus, n (%)**	10 (50%)	4 (20%)	0.096
**Pre-LT Cardiologic Disease, n(%)**	4 (20%)	-	0.106
**Median pre-eGFR (mL/min/1.73m** ^**2**^ **)**	49±9	34±12	0.001
**MELD score**	20 (±7)	26 (±9)	0.065
**Cold ischemia time (min)**	325±85	370±96	0.070
**Warm ischemia time (min)**	45±19	39±10	0.254
**Intraoperative transfusion**			
Red blood cells (Unit)	5 (0-26)	6 (4-11)	0.060
Fresh Frozen Plasma (Unit)	2 (0-18)	8 (0-16)	0.003
Platelets (Unit)	0 (0-10)	2 (0-20)	0.068
**Piggy-back with portacaval shunt**	11 (55%)	17 (85%)	0.082
**Hospital Stay (days)**	20 (11-90)	15 (10-114)	0.242

NASH, nonalcoholic steatohepatitis; eGFR, estimated glomerular filtration rate, and MELD; model for end-stage liver disease.

**Table 2:** Results of secondary endpoints.

	**ATG Group (n=20)**	**BAS group (n=20)**	**p-value**
**One-year patient and graft survival**	95%	95%	1
**Infection **	6 (30%)	7 (35%)	0.510
(i) Cholangitis (gram-negative bacteria)	3	1	
(ii) Diarrheas (Clostridium difficile)	3	-	
(iii) Pneumonia (Klebsiella pneumoniae)	-	2	
(iv) Urinary tract infection (E. coli)	-	2	
(v) MRSA infection (central vein catheter)	-	1	
(vi) Oral Candidiasis	-	1	
**CMV infection **	9 (45%)	7 (35%)	0.519
**Adverse events related to ATG**			
Thrombocytopenia	3 (15%)		
Thrombocytopenia + Leukopenia	1 (5%)		

MRSA, methicillin-resistant Staphylococcus aureus.

## Data Availability

The data used to support the findings of this study are included within the article.

## Abbreviations

ACR: Acute cellular rejection
ATG: Anti-human T-lymphocyte globulin
BPAR: Biopsy-proven acute rejection
CMV: Cytomegalovirus
CNI: Calcineurin inhibitors
eGFR: Estimated glomerular filtration rate
HCV: Hepatitis C virus
ICU: Intensive care unit
i.v.: Intravenously
LT: Liver transplantation
MELD: Model for end-stage liver disease
MMF: Mycophenolate mofetil
PCR: Polymerase chain reaction
TAC: Tacrolimus.
